# Case Report: Concomitant presence of two STIs in a male patient

**DOI:** 10.12688/f1000research.134667.2

**Published:** 2024-06-12

**Authors:** Kaveri Rusia, Bhushan Madke, Yash Kashikar

**Affiliations:** 1 Department of Dermatology, Venereology and Leprosy, Jawaharlal Nehru Medical College, Datta Meghe Institute of Higher Education and Research, Wardha, Maharashtra, 442001, India

**Keywords:** Syphilis, genital warts, HPV, treponema, STI

## Abstract

**Background:**

The spirochaete *Treponema pallidum subsp. pallidum*, which causes the infectious disease syphilis, can be spread through sexual contact or perinatal transmission. In recent years, cases of syphilis have increased, especially among individuals engaging in behaviour that makes them more vulnerable (condomless sex and multiple sexual partners). Condylomata acuminata (external genital warts) is one of the most common viral sexually transmitted infections (STIs). Individuals who are behaviourally vulnerable are also highly prone to two or more STIs. Our case exemplifies the occurrence of two STIs in a young man who was behaviourally vulnerable to acquiring STIs.

**Case:**

We report a case of a 21-year-old year old heterosexual man presenting with concomitant primary syphilis and genital warts. He presented with a painless genital ulcer and warty growths on his glans penis. Examination showed a painless indurated ulcer and multiple genital warts. Serology was positive for quantitative Venereal disease research laboratory test (1:16 titre). The patient was diagnosed with two concomitant STIs. He was treated as per the latest Centers for Disease Control and Prevention (CDC) guidelines for primary syphilis and podophyllin resin for genital warts. After four weeks, the genital ulcer showed complete healing and there was a significant reduction of genital warts.

**Conclusions:**

Individuals with multiple sexual partners engaging in sexual activity without the use of prevention tools are at a greater chance of acquiring two or more STIs. To reduce concomitant transmission, preventive measures against genital ulcer diseases like syphilis, herpes, and chancroid, such as early identification and treatment, and condom distribution, must be strengthened as part of national STI prevention. Patients with two or more STIs should be followed regularly to assess the progress of infection and should be offered timely medical treatment.

## Introduction

Genital ulcer diseases (GUDs) are breaks in the skin and mucosal continuity in the genital and perigenital region, usually resulting from sexually transmitted infections (STIs). Syphilis is a multisystemic, multistage, chronic illness with a varied prognosis and myriad of clinical presentations. ^1^ Anogenital warts, are also called condyloma acuminata, are one of the most common STIs in the developed world, with a frequency of 2.4 infections per 1,000 people per year. ^2^^,^^3^ Individuals engaging in behaviour that makes them more vulnerable (sex without the use of prevention tools and multiple sexual partners) are at increased likelihood of acquiring two or more STIs. Presence of one STI increases the likelihood for acquiring another STI and our case exemplifies the aforementioned phenomenon. Our case presented with two concomitant STIs, one being bacterial and the other being viral in aetiology.

## Case report

**Figure 1.**
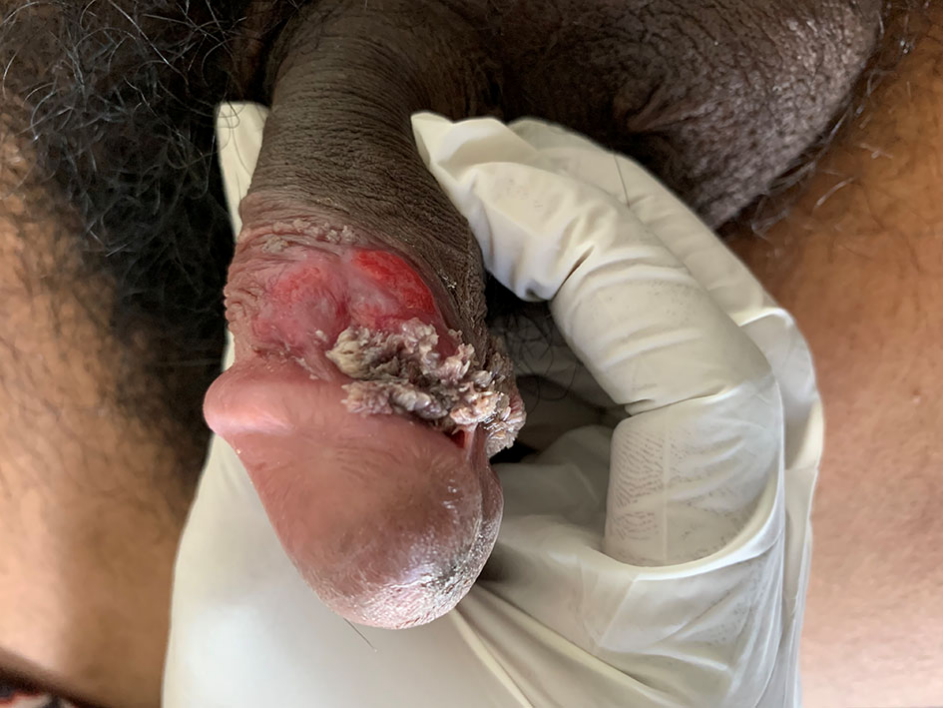
A single clean ulcer on the coronal sulcus.

**Figure 2.**
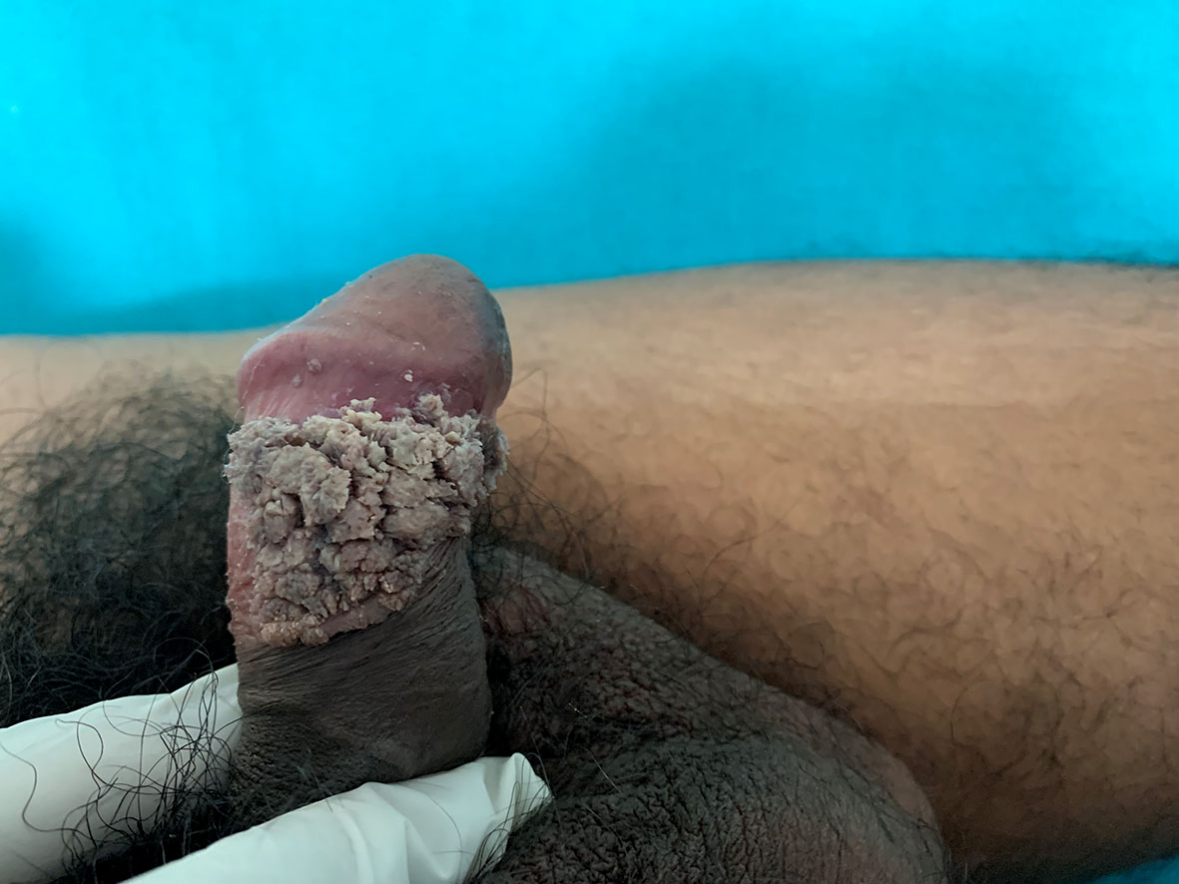
Whitish pink growth on the coronal sulcus.

A 21-year-old male resident of Central India studying at a local college presented to the Dermatology Outpatient Department of the Datta Meghe Institute of Higher Education and Research affiliated tertiary care teaching hospital at Sawangi, Wardha, Maharashtra with complaints of a painless genital ulcer and warty growths on his penis. He reported that the warty lesions had been present for the past two months and the ulcerative lesion appeared three weeks ago. Detailed sexual history revealed regular, penile–vaginal intercourse without the use of prevention tools with sex workers (SWs) for the past six months in his home town, with the last occurrence being approximately four weeks before he presented to our hospital. His general physical examination was within normal limits. There was no history of burning micturition and pus discharge through the urethra. There was a single, painless, indurated ulcer of 3×3 cm in size with rolled edges and minimal discharge on the penis at the coronal sulcus ( Figure 1). Glans penis showed cauliflower floret-like growths on the coronal sulcus and sub-preputial area of the penis ( Figure 2). There was no regional lymphadenopathy. Detailed muco-cutaneous examination of the oral cavity, perianal area and palms and soles were normal. The quantitative Venereal Disease Research Laboratories (VDRL) test was reactive in the titre of 1:16, however the test for treponema pallidum haemagglutination (TPHA) was non-reactive. Serological tests for hepatitis B virus, hepatitis C virus and human immunodeficiency virus (HIV) were negative. On the basis of sexual history, temporal relation, clinical examination and serology, we made a diagnosis of concomitant STIs of primary syphilis and genital warts.

**Figure 3.**
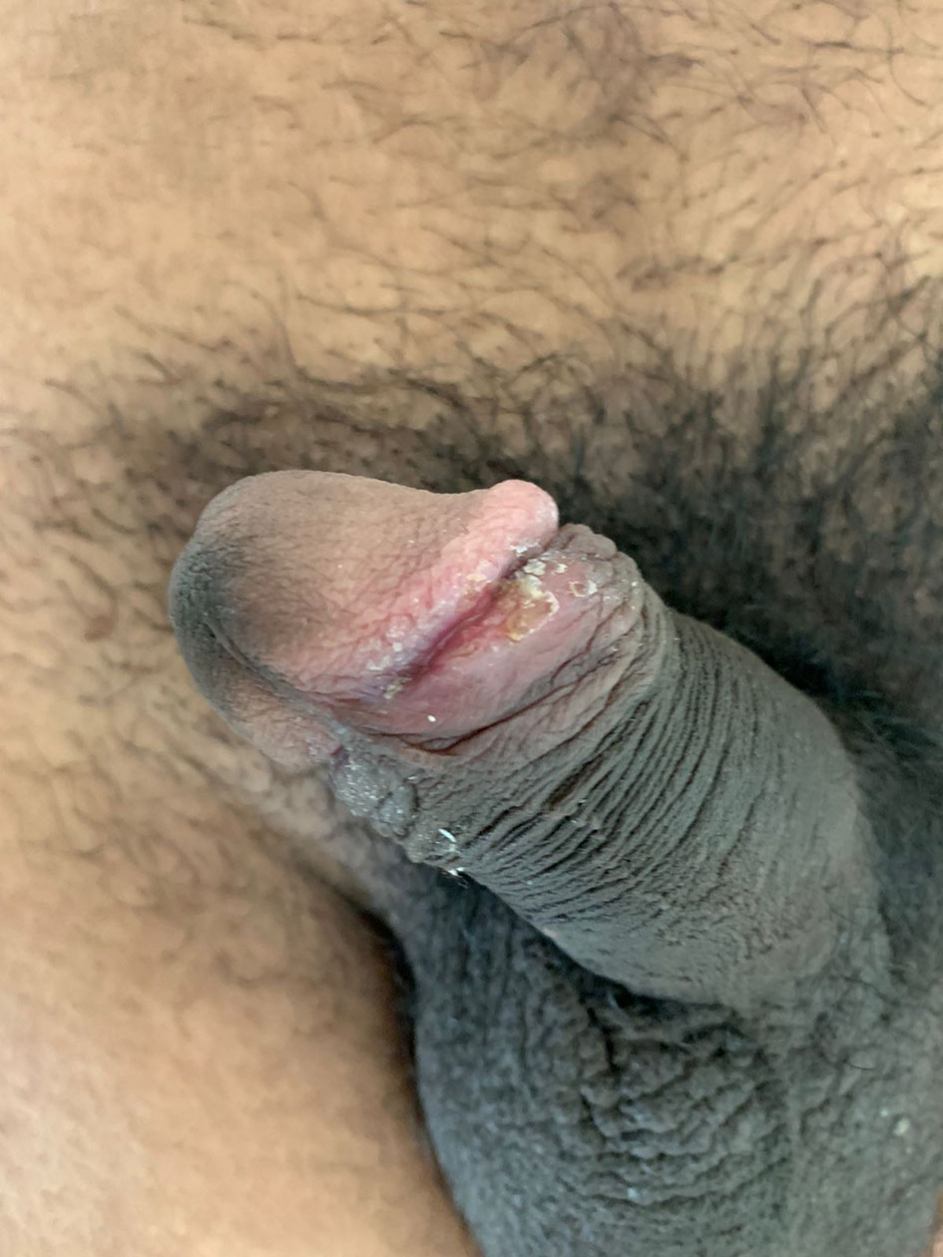
Resolution of genital ulcer and wart after receiving single dose of injectable benzathine penicillin and after application of podophyllin resin at day 30.

We treated the patient with office-based topical application of podophyllin resin (20% w/v) in benzoin (10% w/v) on the genital warts, while the surrounding healthy skin area was protected with petrolatum. Applications were carried out every 10 days until complete clearance of warty lesions. Primary syphilis was treated with a two intramuscular injection of benzathine penicillin (2.4 million units, 1.2 million units in each buttock) after the sensitivity test. Contact tracing is being attempted for the sexual partners for the past three months. On a follow-up visit, the lesion of primary syphilis and genital warts had completely resolved ( Figure 3).

## Discussion

Syphilis is a disease caused by the bacteria *Treponema pallidum* that has a myriad of clinical presentations and is referred to as a “great mimicker” in clinical medicine. Immune evasion and invasiveness are two important pathogenic traits of *Treponema pallidum.* ^4^^–^^7^ Wu *et al.*, ^7^conducted a study where it was found that there is a higher prevalence of syphilis among individuals living with HIV, especially among men who have sex with men. The presence of one STI increases the likelihood that the individual will acquire another STI. The presence of genital ulcer disease increases the risk of acquiring HIV due to mucosal damage and the pool of inflammatory cells at the site of ulcers. ^7^

In a study by Kops *et al.*, it was shown that there are higher chances of acquiring human papilloma virus (HPV) if an individual has an STI. ^6^ A history of prior STI leads to decreased clearance of HPV load and provides an easy access for viral entry into the damaged epithelial barrier. The various factors associated with increased likelihood of concomitant STIs are smoking, substance use disorder and men having sex with men. ^7^

The presence of concomitant STIs suggests the person is behaviourally vulnerable. Individuals with multiple STIs should be investigated for the presence of other venereal transmitted diseases, particularly HIV and hepatitis B virus infection, and appropriate laboratory work-up should be done to confirm the diagnosis.

The primary take-away lesson from our case is as follows: individuals with multiple sexual partners and involved in sexual activity without the use of prevention tools are at greater chance of acquiring two or more STIs. Attempts should be made to perform partner tracing of such cases and individuals should be offered counselling and appropriate medical management. Patients with two or more STIs should be followed regularly to assess the progress of infection and should be offered timely medical treatment.

## Consent

Written informed consent for publication of their clinical details and clinical images was obtained from the patient.

## Data Availability

All data underlying the results are available as part of the article and no additional source data are required.
