# Supplementary material for: Case Report: Concomitant presence of two STIs in a male patient
Source: F1000Res. 2024 Jun 12;12:706. Originally published 2023 Jun 20. [Version 2] doi: 10.12688/f1000research.134667.2 (PMC11329861; doi:10.12688/f1000research.134667.2)
Supplement: Peer review history

# Reviewer report: Reviewer response for version 2

**DOI:** 10.5256/f1000research.167723.r310539

**Published:** 2024-08-22

**Authors:** Giulia Ciccarese

**Affiliations:** 1 Unit of Dermatology, Department of Medical and Surgical Sciences, University of Foggia, Foggia, Italy

The case is interesting and deals with a very important topic, the frequent concomitance of multiple sexually transmitted infections (STIs). However, several details are lacking. Was the patient tested for other sexually transmitted infections, as Neisseria gonorrhoeae, Mycoplasmas , Chlamydia trachomatis and others? Indeed, several studies found that several STIs can be acquired simultaneously, therefore a patient who receives an STI diagnosis should be tested for other silent STIs. See for example: 

 Ref 1, Ref 2.

Are enough details provided of any physical examination and diagnostic tests, treatment given and outcomes?

No

Is the case presented with sufficient detail to be useful for other practitioners?

No

Is sufficient discussion included of the importance of the findings and their relevance to future understanding of disease processes, diagnosis or treatment?

No

Is the background of the case’s history and progression described in sufficient detail?

No

Reviewer Expertise:

Skin infections

I confirm that I have read this submission and believe that I have an appropriate level of expertise to confirm that it is of an acceptable scientific standard, however I have significant reservations, as outlined above.

## References

[1] : Monkeypox outbreak in Genoa, Italy: Clinical, laboratory, histopathologic features, management, and outcome of the infected patients. *J Med Virol* .2023;95(2) : 10.1002/jmv.28560 e28560 10.1002/jmv.28560 36757085

[2] : Sexually transmitted coinfections among at-risk HIV-positive MSM: implications for optimal preemptive treatment. *Front Med (Lausanne)* .2024;11: 10.3389/fmed.2024.1328589 1328589 10.3389/fmed.2024.1328589 38560383 PMC10978595

# Reviewer report: Reviewer response for version 2

**DOI:** 10.5256/f1000research.167723.r310534

**Published:** 2024-08-16

**Authors:** David Šmajs, Eliška Vrbová

**Affiliations:** 1 Department of Biology, Faculty of Medicine, Masaryk University, Brno, Czech Republic; 2 Masaryk University Faculty of Medicine, Department of Biology, Brno, South Moravian Region, Czech Republic

The authors in their report described a case of 21-year-old man diagnosed simultaneously with genital warts and primary syphilis. This case underlines importance of syphilis testing even in the presence of other STIs, and also the need for the follow-up of patient and tracing sexual contacts. Case report is sufficiently described and suitable for indexing

 1) Introduction, line 3: „condyloma acuminata“ should be corrected to „condylomata acuminata“ as it is in Abstract.

 2) Did you repeat the TPHA later to confirm the diagnosis or to see the disease development after treatment? Were any serological tests repeated during follow-up?

Are enough details provided of any physical examination and diagnostic tests, treatment given and outcomes?

Partly

Is the case presented with sufficient detail to be useful for other practitioners?

Yes

Is sufficient discussion included of the importance of the findings and their relevance to future understanding of disease processes, diagnosis or treatment?

Yes

Is the background of the case’s history and progression described in sufficient detail?

Partly

Reviewer Expertise:

Treponemes, treponematic diseases

We confirm that we have read this submission and believe that we have an appropriate level of expertise to confirm that it is of an acceptable scientific standard.

# Reviewer report: Reviewer response for version 2

**DOI:** 10.5256/f1000research.167723.r290390

**Published:** 2024-07-03

**Authors:** Electra Nicolaidou

**Affiliations:** 1 1st Department of Dermatology and Venereology, National and Kapodistrian University of Athens, Athens, Greece

Thank you for answering most of my comments. The first comment, however, as well as the 6th, were not followed by changes in the text. The authors may want to explain to the readers why the used podophillin for the treatment of genital warts even though it is not the most appropiate treatment, according to the guidelines.

Are enough details provided of any physical examination and diagnostic tests, treatment given and outcomes?

Yes

Is the case presented with sufficient detail to be useful for other practitioners?

Yes

Is sufficient discussion included of the importance of the findings and their relevance to future understanding of disease processes, diagnosis or treatment?

Yes

Is the background of the case’s history and progression described in sufficient detail?

Yes

Reviewer Expertise:

I am a dermatologist-venereologist with a special interest in sexually transmitted infections, psoriasis and vitiligo.

I confirm that I have read this submission and believe that I have an appropriate level of expertise to confirm that it is of an acceptable scientific standard, however I have significant reservations, as outlined above.

# Response

**Published:** 2024-07-09

**Authors:** Kaveri Rusia

**Affiliations:** dermatology, datta meghe institute of higher education and research, wardha, maharashtra, India

Thank you for your response. 

 Author response Review Report Point 1:

 I have used podophyllin as it is easily available in our setting.

 Author response Review Report Point 6:

 I have made the necessary changes in version 2 as suggested by you for comment 6.

# Reviewer report: Reviewer response for version 1

**DOI:** 10.5256/f1000research.147742.r266151

**Published:** 2024-05-07

**Authors:** Alessandra Latini

**Affiliations:** 1 San Gallicano Dermatological Institute IRCCS, Rome, Italy

The paper, Concomitant presence of two STIs in a male patient, by Rusia K et al  describes the case of a young heterosexual student who presents with two sexually transmitted infections at the same time as ghenital warts and syphilis. The AA underline how condylomatosis is very common in sexually active young people while the epidemiology of syphilis describes higher prevalence curves in MSM and people with HIV infection.

 The case is well described and documented, the diagnostic and therapeutic process well described. The message that emerges is that syphilis should always be considered in the presence of another STI such as condylomatosis, even if genital warts have notoriously a high incidence in the general population. The authors also highlight the importance of HIV testing and contact tracing in at risk patients.

 (Ref-1). According to the ECDC, a large proportion of syphilis diagnoses in 2022 has been reported in subjects aged 45 years or older. However, while data on increasing STI rates among men who have sex with men have been increasingly reported, a scarce attention has been paid to populations with a lower sexual risk, such as heterosexual individuals.

 This paper considers the risk of syphilis in a heterosexual patient in line with the most recent epidemiological considerations.

Are enough details provided of any physical examination and diagnostic tests, treatment given and outcomes?

Yes

Is the case presented with sufficient detail to be useful for other practitioners?

Yes

Is sufficient discussion included of the importance of the findings and their relevance to future understanding of disease processes, diagnosis or treatment?

Yes

Is the background of the case’s history and progression described in sufficient detail?

Yes

Reviewer Expertise:

STIs

I confirm that I have read this submission and believe that I have an appropriate level of expertise to confirm that it is of an acceptable scientific standard.

## References

[1] STI cases on the rise across Europ. *European Centre for Disease Prevention and Control* . Reference source

# Reviewer report: Reviewer response for version 1

**DOI:** 10.5256/f1000research.147742.r235347

**Published:** 2024-02-15

**Authors:** Electra Nicolaidou

**Affiliations:** 1 1st Department of Dermatology and Venereology, National and Kapodistrian University of Athens, Athens, Greece

This is a well written case report of a patient presenting with two concomitant STIs, syphilis and genital warts, that were typical in clinical appearance and responded well to treatment. The case is well presented.

 My comments are as follows:

 1)The patient was treated according to the CDC guidelines for syphilis, but these guidelines were not followed for the treatment of genital warts. These guidelines state that "podophyllin resin is no longer a recommended regimen because of the number of safer regimens available" and that "the area to which treatment is administered should not contain any open lesions, wounds of friable tissue". So, the authors may want to explain why they used this treatment for the genital warts, especially in a patient with an ulcer surrounding part of the warts.

 2)Abstract, Background: MSMs have high rates of syphilis because they often engage in behavior that makes them more vulnerable, so there is no need to refer to them separately.

 3) Abstract, Background: "be exposed to one or more STIs" could be changed to "two or more STIs"

 4) Abstract, Case: "quantitative serological disease research laboratory test" could be changed to "quantitative Venereal disease research laboratory test"

 5) Abstract, Conclusions: Human papillomavirus is not correctly spelled and it does not cause genital ulcer diseases

 6) Introduction: Anogenital warts are also called condylomata acuminata (not condyloma acuminatum, which is singular number)

 7) Case report: "Primary syphilis was treated with a single intramuscular injection..." It is actually two injections, not a single one, as it is correctly described by the authors in the brackets.

Are enough details provided of any physical examination and diagnostic tests, treatment given and outcomes?

Yes

Is the case presented with sufficient detail to be useful for other practitioners?

Yes

Is sufficient discussion included of the importance of the findings and their relevance to future understanding of disease processes, diagnosis or treatment?

Yes

Is the background of the case’s history and progression described in sufficient detail?

Yes

Reviewer Expertise:

I am a dermatologist-venereologist with a special interest in sexually transmitted infections, psoriasis and vitiligo.

I confirm that I have read this submission and believe that I have an appropriate level of expertise to confirm that it is of an acceptable scientific standard, however I have significant reservations, as outlined above.

# Response

**Published:** 2024-02-15

**Authors:** Kaveri Rusia

**Affiliations:** dermatology, datta meghe institute of higher education and research, wardha, maharashtra, India

Thank you for your responses. Necessary changes can be done according to your comments.

 I have used podophyllin as it is easily available in our setup.
